# Obesity, Inflammation, Toll-Like Receptor 4 and Fatty Acids

**DOI:** 10.3390/nu10040432

**Published:** 2018-03-30

**Authors:** Marcelo Macedo Rogero, Philip C. Calder

**Affiliations:** 1 Nutritional Genomics and Inflammation Laboratory, Department of Nutrition, School of Public Health, University of São Paulo, 01246-904 São Paulo, Brazil; 2 Food Research Center (FoRC), CEPID-FAPESP, Research Innovation and Dissemination Centers São Paulo Research Foundation, São Paulo 05468-140, Brazil; 3 Human Development and Health Academic Unit, Faculty of Medicine, University of Southampton, Southampton SO16 6YD, UK; P.C.Calder@soton.ac.uk; 4 National Institute for Health Research Southampton Biomedical Research Centre, University Hospital Southampton National Health Service Foundation Trust and University of Southampton, Southampton SO16 6YD, UK

**Keywords:** inflammation, toll-like receptor 4, obesity, fatty acids

## Abstract

Obesity leads to an inflammatory condition that is directly involved in the etiology of cardiovascular diseases, type 2 diabetes mellitus, and certain types of cancer. The classic inflammatory response is an acute reaction to infections or to tissue injuries, and it tends to move towards resolution and homeostasis. However, the inflammatory process that was observed in individuals affected by obesity and metabolic syndrome differs from the classical inflammatory response in certain respects. This inflammatory process manifests itself systemically and it is characterized by a chronic low-intensity reaction. The toll-like receptor 4 (TLR4) signaling pathway is acknowledged as one of the main triggers of the obesity-induced inflammatory response. The aim of the present review is to describe the role that is played by the TLR4 signaling pathway in the inflammatory response and its modulation by saturated and omega-3 polyunsaturated fatty acids. Studies indicate that saturated fatty acids can induce inflammation by activating the TLR4 signaling pathway. Conversely, omega-3 polyunsaturated fatty acids, such as eicosapentaenoic acid and docosahexaenoic acid, exert anti-inflammatory actions through the attenuation of the activation of the TLR4 signaling pathway by either lipopolysaccharides or saturated fatty acids.

## 1. Obesity

Obesity is a multifactorial and polygenic condition that has become a very concerning public health issue that is affecting both developed and developing countries [1,2,3]. Overweight individuals (defined as body mass index (BMI) ≥ 25 kg/m^2^) account for approximately 30% of the global population, i.e., 2.1 billion people, of whom more than 600,000 are classified as obese (defined as BMI ≥ 30 kg/m^2^) [4]. The analysis conducted by the Global Burden of Disease Study 2013 showed that the overweight prevalence increased to 27.5% of adults and 47.1% of children in the past three decades [5]. The prevalence of obesity is currently higher in developed countries; nevertheless, approximately two-thirds of the obese population lives in developing countries [6]. Based on the current scenario, it is estimated that up to 50% of the global population will be classified as overweight or obese by 2030 [7]. Approximately 35% of adult individuals and 17% of children and adolescents (2 to 19 years old) are considered to be obese (defined by values above the 95th percentile of the BMI curve of these age groups) in the United States. It is estimated that approximately 300,000 people die due to obesity in the United States (U.S.) every year, which is the second highest cause of preventable death [8]. 

Cardiovascular diseases, type 2 diabetes (DM2), non-alcoholic fatty liver disease, and cancer stand out among the main health issues that are responsible for morbidity related to the obesity [9]. Obesity treatment and the treatment of its associated complications in developing countries has led to significant cost increases in healthcare. Costs that are linked to DM2, in particular, stand out, since 20–30% of overweight people present with a DM2 diagnosis, while 85% of diabetic patients are overweight or obese [10]. Calle et al. [11] conducted a prospective study of more than one million men and women and found that the lowest mortality rates, for all causes, in both men and women, occur in individuals with BMIs that are between 23.5 and 24.9 and 22.00 and 23.4 kg/m^2^, respectively. Another study including 900,000 adult individuals found that BMIs that were above 25 kg/m^2^ were associated with a 30% increase in general mortality rate per each 5 kg/m^2^ increase [12].

Obesity results from the interactions of different factors, including genetic, metabolic, behavioral, and environmental ones. Accordingly, the dramatic increase in obesity prevalence rates suggests that behavioral and environmental components are the main factors that are responsible for obesity, with an emphasis on eating habits and exercise. With regard to eating, modern societies converge to an eating pattern called the Western diet, which is characterized by the intake of foods with high energy densities. Such densities derive from the high contents of fat and carbohydrate, especially sugars, that are found in these food types, a fact that contributes to obesity development [13,14].

The profile of fatty acids that are present in a diet may also be relevant to obesity. It is worth highlighting that, according to anthropological and epidemiological studies, humans from the Paleolithic Era—40,000 years ago—consumed a ratio of omega-6 (ω-6) to omega-3 (ω-3) polyunsaturated fatty acids of approximately 1, mainly due to a high intake of marine and vegetable sources of ω-3 polyunsaturated fatty acids (PUFAs). However, there was a significant increase in the intake of lipids, *trans* fatty acids, and ω-6 PUFAs after the Industrial Revolution, as well as a small increase in the intake of ω-3 fatty acids; meanwhile, intakes of vitamins C and E decreased. Such changes are particularly relevant if one takes into account the participation of these nutrients in the inflammatory response, which is linked to the physiopathology of different non-transmissible chronic diseases, such as obesity, DM2, cardiovascular diseases, hypertension, and cancer [15,16,17]. 

## 2. Inflammation, Adipose Tissue and Obesity

Inflammation is a central component of innate immunity, and microorganism destruction is the prime function of the inflammatory response, which is a process that involves the participation of effector cells in contact with pathogens that are living in the infected tissue. Microbial components, such as lipopolysaccharides (LPS) that are found in the cell wall of Gram negative bacteria, can trigger an inflammatory response through their interactions with cell-surface receptors found, for instance, in cells from the immune system, such as macrophages and neutrophils. Inflammation in response to microorganisms involves the increased synthesis and secretion of a number of mediators, including chemokines and cytokines. The latter include tumor necrosis factor (TNF)-α and interleukin (IL)-1, which act on endothelial cells and leukocytes to promote the recruitment and activation of leukocytes in the inflammatory area [18,19]. 

Inflammation can be classified as acute or chronic. Acute inflammation presents via three principal components: (i) changes in the vascular caliber, which result in increased blood flow in the inflammatory focus; (ii) structural changes in the microcirculation, which favor the exit of plasma proteins and leukocytes from the blood to the tissue; and, (iii) adhesion and transmigration of leukocytes from the microcirculation to the tissue, as well as their further activation, which allows the elimination of harmful agents. As soon as the infection is eliminated, or at least controlled, mechanisms are activated that act to limit any type of aggression against the host and to initiate the tissue repair process. Such a process aims to reduce the inflammation and it is termed resolution. Resolution is now known to be an active process involving the activation of negative feedback mechanisms, such as anti-inflammatory cytokine secretion, a reduction in receptor expression, activation of regulatory cells, and the production of pro-resolving lipid mediators [20,21,22]. 

Histamine, bradykinin, neuropeptides, prostaglandins, thromboxanes, leukotrienes, and platelet-activating factor stand out among the non-cytokine/chemokine mediators that are involved in the inflammatory response. The generation of eicosanoids initially occurs due to activation of phospholipase A2, which hydrolyzes membrane phospholipids to yield a free fatty acid. Arachidonic acid, an ω-6 PUFA, is predominant among the fatty acids released by phospholipase A2. The released fatty acids are used as a substrate by the cyclooxygenase enzymes (COX), which catalyze the synthesis of prostaglandins and thromboxanes, as well as by lipoxygenase (LOX) enzymes, which catalyzes the synthesis of leukotrienes. Such mediators are responsible for many aspects of the inflammatory response, such as vasodilation (prostaglandin E_2_) and leukocyte migration (leukotriene B_4_) [23,24,25].

Chronic inflammation involves the progressive changes in inflammatory cells as well as in tissue destruction and repair due to the on-going inflammatory process. Accordingly, inflammation can become pathologic because of the loss of tolerance or regulatory processes. As a result, there is an increase the plasma concentrations of many inflammatory biomarkers and in the number of activated inflammatory cells in the bloodstream as well as in the primary lesion area. Such changes can be easily observed, for instance, in patients with frank chronic conditions, like rheumatoid arthritis and inflammatory bowel diseases [26,27].

**Figure 1:**
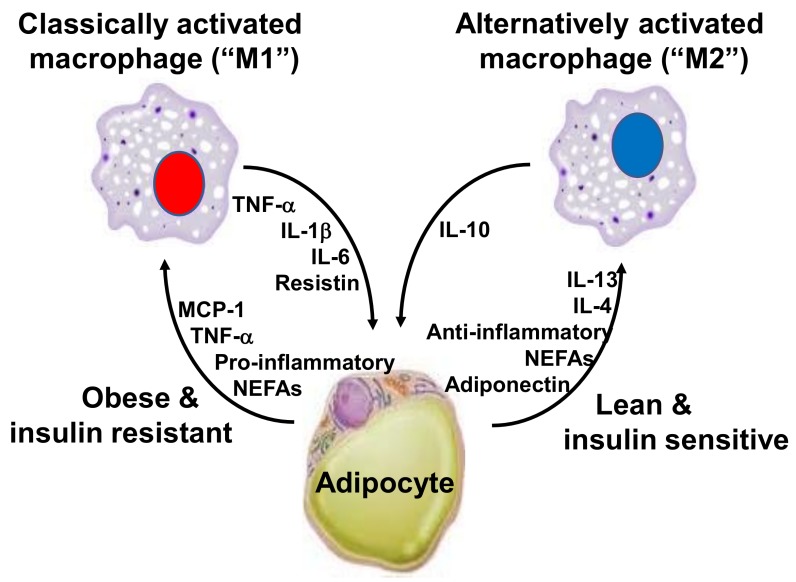
Interaction between M1 and M2 macrophages and adipocytes. Abbreviations: IL, interleukin; MCP, monocyte chemotactic protein; NEFAs, non-esterified fatty acids; TNF, tumor necrosis factor.

Chronic inflammation can also be present at lower intensities than has been seen in the classic inflammatory diseases. Evidence that obesity results in inflammation started emerging in the 1990s. This inflammation is directly involved in the etiology of cardiovascular diseases, DM2, and certain cancer types [28]. Hotamisligil et al. [29] found that genetically obese rodents, such as db/db and ob/ob mice and fa/fa rats, had increased expression of the TNF-α gene in white adipose tissue. They identified that the neutralization of TNF by anti-TNF-α antibodies mitigated the resistance of these animals to insulin action, establishing a link between inflammation, insulin resistance, and hyperglycemia. Macrophages from the stromal vascular fraction of adipose tissue appear to be the main cell type that is responsible for TNF-α and IL-6 release from the adipose tissue. The increased concentration of cytokines in this tissue is mostly derived from the infiltration of M1 macrophages, which are activated in the classical way and are characterized by the high expression of pro-inflammatory cytokines, like TNF-α, IL-1β and IL-6 [30,31,32] (Figure 1). It should be noted that macrophages correspond to about 40% of total white adipose tissue cells in obese mice and humans, as compared to only 18% in lean controls [33]. In the white adipose tissue, the expression of monocyte chemoattractant protein (MCP)-1 correlates positively with adiposity, and it is also higher in visceral adipose tissue when compared to subcutaneous adipose tissue [34,35]. The receptor for MCP-1, C-C chemokine receptor type 2 (CCR2), is expressed on monocytes present in peripheral blood and on adipose tissue macrophages. This implies that obesity favors the process of migration of blood monocytes into the visceral adipose tissue of obese individuals, which then differentiate into macrophages. This process is regulated by colony stimulating factors, such as macrophage-specific growth factor, called colony stimulating factor 1 (CSF-1) or macrophage colony-stimulating factor (M-CSF) [36].

In mammals, there are two types of adipose tissue: white and brown adipose tissue (BAT). BAT is specialized in the production of heat (thermogenesis) and, therefore, actively participates in the regulation of body temperature. BAT deposits are found in fetuses and newborns. In adult humans, there is a small volume of BAT in the cervical supra-clavicular, supra-adrenal, and para-spinal regions [37,38]. Brown and white adipocytes appear to have different physiology and opposing functions [39] Beiging/browning of white adipose tissue promotes energy expenditure by triggering thermogenesis, which suppresses diet-induced weight gain, as well as enhancing the efficiency of BAT activity [40]. In this context, individuals with low amounts of BAT would be prone to the development of obesity. Studies in animals lacking BAT or uncoupling protein 1 (UCP1) have clearly demonstrated the involvement of BAT thermogenesis in the protection against diet-induced obesity (DIO) [41]. Decreasing BAT activity or the removal of BAT in mice provokes increased glycemia and plasma triglyceride concentration and promotes insulin resistance [42]. Also, in humans, BAT activity was found to be inversely related to BMI and fat mass [43]. Furthermore, visceral adipose tissue inflammation may also be linked to the lower BAT volume, since TNF-α has been shown to induce brown adipocyte apoptosis and to hamper BAT differentiation [44]. 

Obesity is a relevant causal factor in the etiology of insulin-action resistance. Thus, obese patients present with reduced insulin action in the skeletal muscle due to lower phosphorylation of the tyrosine residues of the insulin receptor substrate (IRS)-1 and the reduced phosphatidylinositol-4,5-bisphosphate 3-kinase (PI3K) activity in this tissue. Such an outcome can cause a further reduction in insulin-induced glucose transport into the muscle tissue [45].

An increased inflammatory response is an important factor in the etiology of insulin-action resistance in obese patients. Such a response triggers the activation of protein kinases related to Toll signaling pathways and TNF-α receptors, such as the inhibitor of kappa B kinase (IKK) and c-jun N-terminal kinase (JNK)-1, which are capable of phosphorylating IRS-1 at the serine 307 residue. This reduces IRS-1 interaction with the insulin receptor beta subunit, and, consequently, causes decreased insulin signal transduction [46,47]. JNK knockout mice show lower adiposity, enhanced sensitivity to insulin and an increased capacity for insulin receptor signaling even when they are fed a lipid-rich feed. These findings suggest that activation through JNK is an important mechanism linked to insulin resistance in obese patients [48].

Among the inflammatory biomarkers that are related to obesity, IL-6 favors insulin-action resistance in obese individuals due to the induction of the cytokine signaling suppressor protein 3 (SOCS3), which physically associates itself with tyrosine phosphorylated proteins, such as the insulin receptor. In addition, SOCS3 decreases the phosphorylation of IRS-1 tyrosine, which weakens the IRS-1 coupling to the insulin receptor and the subsequent association between IRS-1 and phosphatidylinositol-3 kinase (PI3K). These findings suggest that SOCS3 is a relevant inhibitor of the insulin signaling pathway, as well as allowing a better understanding of the IL-6 effect on the insulin-action resistance that is induced by obesity [49]. 

Understanding that the immune system and different metabolic pathways are closely related to each other, as well as that they are functionally dependent, is essential for studies that are focused on obesity and on its possible metabolic repercussions. Thus, signaling pathways that are responsive to nutrient intake and the presence of pathogens are evolutionarily conserved and greatly integrated [50]. The excessive intake of obesity-associated nutrients can be detected by innate recognition receptors, and this results in the activation of pro-inflammatory signaling pathways as well as in stress responses in many parts of the body. This causes low-intensity chronic inflammation, defined by Hotamisligil et al. [30] as metabolic inflammation or as meta-inflammation, which is different from the classic inflammatory response. Moreover, the genesis of this inflammation is closely related to lifestyle and mainly to the quality of diet and exercise [51]. 

Meta-inflammation development is associated with a wide and integrated network of intracellular signal pathways, among which inhibitor of nuclear factor kappa-B kinase subunit beta (IKK-β) and c-Jun N-terminal kinase 1 (JNK-1) stand out. These proteins induce the synthesis of inflammatory mediators in different cell types. IKK-β and JNK-1 activation results in activating the transcription factors nuclear factor kappa B (NF-κB) and the activating protein (AP)-1, which translocate to the cell nucleus and activate the transcription of many genes encoding the proteins that are involved in inflammation, including TNF-α and COX-2. This process allows for the continuity of the inflammatory reaction, which is associated with conditions, such as atherogenesis and insulin-action resistance [52,53].

This systemic inflammatory response mainly originates from adipose tissue, which produces a wide variety of pro-inflammatory cytokines and chemokines, called adipokines [23]. However, currently, it is known that there are other tissues involved in meta-inflammation, such as the liver [54], pancreas [55], hypothalamus [56,57], and skeletal muscle [58]. It seems likely that the chronic low-grade inflammation that develops in adipose tissue with obesity is “transferred” to these other tissues through the appearance of active inflammatory mediators in the bloodstream. 

In the context of inflammation and obesity, the role of gut microbiota in the development of metabolic disease should be noted. Studies have shown that certain bacteria populations produce enzymes that increase the efficiency of nutrient digestion, leading to an improved nutrient supply to the host, therefore, contributing to increased energy storage in the adipose tissue. The resulting increase in body adiposity can trigger the development of insulin resistance. There is also evidence that the gut microbiome can modulate that genes that are involved in energy storage and expenditure [59,60,61,62].

In 2004, Backhed et al. [61] reported that conventionally reared mice had a 42% increase in body fat and a 47% increases in periepididymal adipose tissue when compared to germ-free mice. Furthermore, transfer of the microbiota from the bowel of the conventional mouse to the gut of the germ-free mouse resulted in a 57% increase in body fat in two weeks, although feed consumption decreased. This result highlights the important role that the intestinal microbiota plays in energy homeostasis and its potential involvement in the etiology of obesity. Germ-free mice are resistant to diet -induced adiposity, which is associated with increased activity of AMP-activated protein kinase (AMPK) in liver and muscle and increased expression of adipose factor that is induced by fasting (Fiaf) in the small intestine [62]. On the other hand, the inoculation of the microbiota of conventional mice fed with this diet into germ-free animals results in an increase in adiposity [59].

It should also be noted that the dysbiosis that is associated with consuming a high-fat diet has been shown to increase intestinal permeability, which results in a greater translocation of LPS from the intestinal lumen to the blood circulation. This metabolic endotoxemia is associated with increased body fat, glucose intolerance, and increased expression of proinflammatory mediators and macrophage infiltration in white adipose tissue [60].

## 3. Toll-Like Receptor 4 and Inflammatory Response

The innate immune systems of mammals—which encompasses cells such as neutrophils and macrophages—use different strategies to recognize microorganisms. One of these strategies is based on recognizing general aspects of molecules associated with pathogens (pathogen-associated molecular patterns, or PAMPs) that result from microbial metabolism that is conserved throughout the evolution of the species. These molecules are widely distributed among pathogens; for instance, the LPS molecule is common in all Gram-negative bacteria, although it is not produced by the host [63,64,65]. 

Innate immune system receptors that are capable of recognizing PAMPs are called pattern recognition receptors, and these induce the expression of pro-inflammatory cytokines—for example, TNF-α and IL-1β—as well as activating the host’s antimicrobial defense mechanisms, such as the synthesis of reactive oxygen and nitrogen species, including hydrogen peroxide and nitric oxide (NO), respectively [66,67]. PAMP recognition can induce cluster of differentiation 80 (CD80) and cluster of differentiation 86 (CD86) costimulatory molecules on the surface of cells, presenting antigens, as well as inducing small antigenic peptides that are linked to major histocompatibility complex (MHC) class II molecules in cell membranes that present antigens to CD4^+^ T lymphocytes so activating adaptive immune responses [68].

The innate immune system recognizes PAMPs through toll-like receptors (TLRs) that are a family of transmembrane proteins that are responsible for playing an essential role in the innate immune system [69]. The main function of the TLR protein lies in controlling inflammatory and immunological responses. TLRs can recognize a whole variety of microbial PAMPs. Eleven different TLRs have been identified in humans and thirteen among all mammals [70]. TLRs belong to the IL-1 receptor (IL-1R) superfamily, which have a significant homology in their cytoplasmic regions, such as in the Toll/IL-1R (TIR) domain. The TIR domain is needed for the interaction and recruiting of many adaptive molecules that are involved in the activation of signaling pathways [67].

TLRs are expressed in different cell compartments and are recognized by many PAMPs deriving from viruses, pathogenic bacteria, fungi, and protozoa. TLR1, TLR2, TLR4, TLR5, TLR6, and TLR11 are expressed in the cellular membrane, whereas TLR3, TLR7, TLR8 and TLR9 are expressed in intracellular compartments, such as the endosome and the endoplasmic reticulum. Based on the amino acid sequence and on the genomic structure, TLRs can be divided into five subfamilies: TLR2, TLR3, TLR4, TLR5, and TLR9. The subfamily TLR2 comprises TLR1, TLR2, TLR6, and TLR10, whereas the subfamily TLR9 encompasses TLR7, TLR8, and TLR9 [71,72,73].

TLR4 was the first TLR reported in humans; it is expressed in innate immune cells, including monocytes, macrophages, and dendritic cells, as well as in other cell types, like adipocytes, enterocytes, and muscle cells. As indicated above, LPS is the primary agonist for TLR4 [74]. LPS is an integral structural component that is found in the external membrane of Gram-negative bacteria as well as representing one of the most powerful microbial inflammation indicators. It is a complex glycolipid composed of one hydrophilic polysaccharide and one hydrophobic domain called lipid A [75]. There is some evidence that saturated fatty acids can also bind to TLR4 and activate TLR4-mediated signaling pathways [76,77]. Also, there are other endogens ligands for TLR4, like heat shock protein (Hsp) 60, Hsp 70, type III repeat extra domain A of fibronectin, oligosaccharides of hyaluronic acid, polysaccharide fragments of heparan sulfate, and fibrinogen [78]. In the context of obesity, the increase in the plasma fibrinogen levels, which represents a positive acute phase protein, acts as a factor that is involved in the activation of the TLR4 pathway, and, consequently, in the amplification of the inflammatory response [79].

The interaction between LPS and TLR4 induces the synthesis of pro-inflammatory cytokines, such as TNF-α, IL-1β, IL-6, IL-8, and IL-12, which, in turn, work as endogenous inflammatory mediators by interacting with receptors found in different target cells. In addition to cytokines, macrophages release a whole variety of biological mediators in response to LPS, including platelet activation factor, prostaglandins, enzymes, and reactive oxygen and nitrogen species, such as superoxide anion and nitric oxide (NO). The synthesis of these pro-inflammatory mediators by monocytes and macrophages is designed to inhibit the growth and the dissemination of pathogens and to eliminate them either directly or through induction of adaptive immune responses [63,80].

LPS initially binds to the LPS-binding protein (LBP), which is found in the blood or in extracellular spaces. This protein promotes LPS binding to the CD14 molecule, which, in turn, is moored to the lipid bilayer by means of a glycophosphatidylinositol group that is found in most cells, except for endothelial ones. CD14 can also exist as a soluble protein, and, in this case, can lead LPS to the cell surface. The CD14 molecule is not found in transmembrane and intracellular domains; thus, it cannot trigger signal transduction processes on its own. When LPS binds to CD14, LBP dissociates itself and the LPS-CD14 complex physically associates with TLR4. Such a receptor needs an additional molecule, the so-called extracellular accessory protein (MD2), which binds to the TLR4 extracellular complex in order to recognize LPS [71]. 

Following ligand binding, TLRs dimerize and undergo conformational changes that are required for the subsequent recruitment of cytosolic TIR domain-containing adaptor molecules, including the cytoplasmic adapter protein MyD88. The association between TLR4 and MyD88 gathers proteins from the IL-1 receptor associated kinase (IRAK) family. Two members (IRAK4 and IRAK1) are phosphorylated in sequence, and this disrupts them from the receptor complex and promotes their association with TNF receptor associated factor 6 (TRAF6). TRAF6 then activates mitogen activated protein kinase (MAPK) proteins. These kinases can activate the AP-1 transcription factor [81]. 

**Figure 2:**
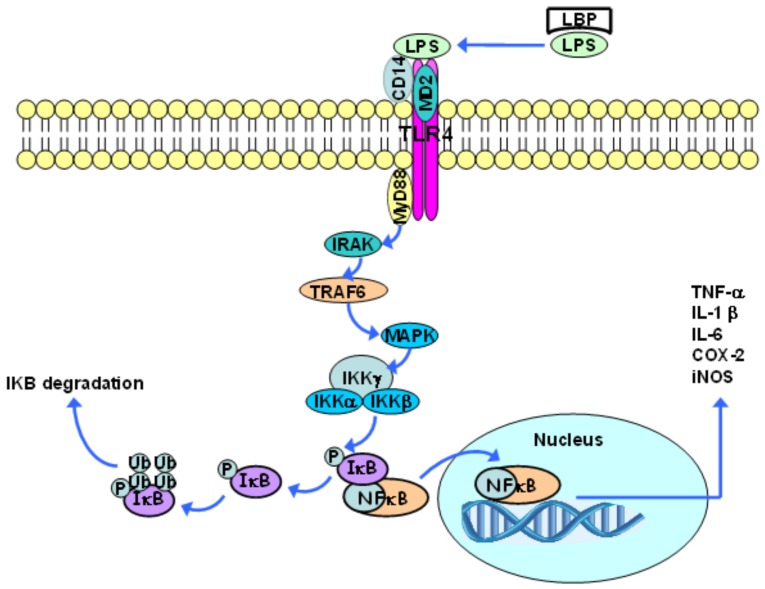
Toll-like receptor 4 (TLR4) induced signaling activates the transcription factor NFκB. LBP: LPS-binding protein; LPS: lipopolysaccharides; IRAK: IL-1 receptor associated kinase; TRAF6: TNF receptor associated factor 6; MAPK: mitogen activated protein kinase; IKK: inhibitor of nuclear factor kappa-B kinase; iNOs: inducible nitric oxide synthase.

The transcription factor NF-κB, which is found in a dimeric form in the cytoplasm of non-stimulated cells, is inactive when it is associated with κB inhibitors (IκB) (Figure 2). The family of IκB proteins includes IκBα, IκBβ, IκBε, and Bcl-3, as well as the carboxy-terminal regions of NF-κB1 (p105) and NF-κB2 (p100). The IκB proteins bind to different NF-κB dimers, although they have different affinities and specificities; therefore, besides the different NF-κB dimers that are found in a specific cell type, there are a large number of combinations of the IκB and the NF-κB dimers [82,83].

Via MAPK, TRAF6 activates the IκB kinase complex (IKK), which is composed of two catalytic subunits (IKKα and IKKβ) and one regulatory subunit (IKKγ), and has the capacity to induce IκB phosphorylation. This phosphorylation results in IκB dissociation from the NF-κB complex and its subsequent polyubiquitination, which, in turn, leads to IkB degradation (mediated by the 26S proteasome) [73,81]. This process allows for the NF-κB dimer to translocate into the nucleus and to activate the transcription of many κB-dependent genes, such as the genes of pro-inflammatory cytokines, including TNF-α, IL-1β, IL-6, COX-2, and inducible nitric oxide synthase (iNOS) (Figure 2). NF-κB also stimulates the synthesis of IκB. Accordingly, the newly synthesized IκB binds to NF-κB and suppresses its activity, providing a feedback inhibition mechanism [74,81]. There are five members of the family of NF-κB transcription factors in mammals: NF-κB1 (p105/p50), NF-κB2 (p100/p52), RelA (p65), RelB, and c-Rel, which can dimerize to form homodimers and heterodimers that, in turn, are associated with specific transcriptional responses to different stimuli. NF-κB1and NF-κB2 do not contain transcriptional activation domains and their homodimers work as repressors. On the other hand, Rel-A, Rel-B, and c-Rel drive the transcriptional activation domain, and, except for Rel-B, are capable of forming homodimers and heterodimers along with other members of this family of proteins. Consequently, the balance between different NF-κB homodimers and heterodimers regulates the transcriptional activity level. It is worth highlighting that these proteins are expressed in a specific cell and tissue pattern, which leads to an additional level of regulation. NF-κB1 (p50) and RelA, for example, are broadly expressed, and, therefore, the p50/RelA heterodimer is the most common NF-κB-binding activity inducer [82,83]. 

Human monocytes express TLR1, TLR2, TLR4, TLR5, TLR6, TLR8, and TLR9; but TLR2 and TLR4 are the receptors that are most commonly expressed in these cells. The expression of TLR2 and TLR4 in the plasma membrane of monocytes has been confirmed by flow cytometry; TLR2 and TLR4-binding (by peptidoglycan and LPS, respectively) generates pro-inflammatory cytokine secretion in these cells. Moreover, TLR2 and TLR4 activation recruits monocytes and forms foam cells in murine models of atherosclerosis [30,84].

Studies that were conducted in vitro with cell cultures showed the negative effects of pro-inflammatory cytokines deriving from TLR4 signal pathway activation on glucose uptake and on the metabolism of fatty acids [33,85,86]. TLR4 gene deletion in mice has a protective effect against adipose tissue inflammation and against the resistance to insulin action that is induced by the intake of a high fat diet, a fact that points towards the causal role played by TLR4 in metabolic changes driven by over-eating and obesity [87,88].

Humans with type I diabetes exhibit a greater expression of TLR2 and TLR4 in the cellular membrane in monocytes, as well as greater MyD88 protein content and IRAK phosphorylation in monocytes in the peripheral blood than in control groups [89]. Individuals with DM2 show increased cellular membrane levels of TLR2 and TLR4 in blood monocytes, as well as a higher concentration of IL-1β, IL-6, IL-8, and TNF-α in serum than in controls [90]. Similarly, TLR2, TLR4, and MyD88 are more highly expressed in blood mononuclear cells and in the abdominal subcutaneous white adipose tissue in obese and diabetic individuals than in patients with normal weight [63,80]. Also, overweight and obese people showed increased expression of TLR2 and TLR4 on peripheral blood mononuclear cells and in adipose tissue in comparison with lean people; the expression levels of TLR2 and TLR4 increased significantly with increasing body mass index [91].

Furthermore, insulin-action resistance in obese individuals can increase the expression of TLR4, which depends on the designated PU.1 transcription factor, which, in turn, regulates the gene expression that is related to the activation and the differentiation of myeloid cells, including the TLR2, TLR4, and TLR9 receptors [92,93]. Insulin has a suppressive effect on the expression of TLR4 and on the activity of the PU.1 transcription factor; however, the suppressive effect of the hormone would be expected to be reduced due to the insulin-action resistance related to obesity. Such a reduction would increase the expression of TLR4 in peripheral blood monocytes [94]. In view of this, it seems that the increase of the inflammatory response favors the occurrence of resistance to the action of the insulin, through the activation of the IKK-β and JNK kinases that reduce the activation of IRS-1 in the insulin signaling pathway. Conversely, the presence of insulin resistance favors the expression of TLR4, suggesting that insulin resistance promotes inflammation.

As described earlier, the TLR4 pathway increases the expression of pro-inflammatory cytokines, such as TNF-α, IL-1, and IL-6, by activating the transcription factors NF-κB and AP-1. These cytokines, in turn, increase the hepatic synthesis of CRP, which is the classic positive acute phase reactant and the most studied and accepted inflammatory biomarker. CRP is often used in clinical practice due to its high stability (mean half-life of 19 hours) and its rapid production in response to inflammatory stimuli [95,96]. It is important to note that other inflammatory biomarkers, such as IL-6, TNF-α, the intercellular adhesion molecule (ICAM)-1, P-selectin, E-selectin, the monocyte chemotactic protein (MCP)-1, fibrinogen, and soluble CD40, have been characterized as predictors of cardiovascular disease, regardless of other cardiovascular risk factors [19,26]. 

Dietary lipids can cause changes in the expression patterns of TLRs [97]. Ingestion of a high calorie (910 kcal), high lipid (51 g), and high carbohydrate (88 g) meal by normal weight individuals caused significant changes in TLR in the post-prandial period, with TLR2 and TLR4 increasing in blood mononuclear cells. This reinforces the potential importance of postprandial inflammation for obesity, DM2, and cardiovascular disease physiopathology [98,99]. A high-fat meal also leads to increased NF-κB activation in the post-prandial period, as well as increased leucocyte activation, as assessed by the surface expression of CD11a, CD11b, and CD62L [100], and metabolic endotoxemia (i.e., increased plasma LPS levels) [101]. 

## 4. Fatty Acids, Toll-Like Receptors and Inflammation

### 4.1. Saturated Fatty Acids

Saturated fatty acids, particularly lauric acid and palmitic acid, are capable of stimulating an inflammatory response through the TLR4 signaling pathway [102]. Lee et al. [103] published the first study that demonstrated the effect of different fatty acids on the TLR4 signaling pathway. In this study, it was verified that lauric, palmitic, and stearic acids could induce COX-2 expression through an NFκB-dependent mechanism in a macrophage cell line. Among the saturated fatty acids that were tested, lauric acid (C12:0) had the greatest activation capacity through TLR4. Different from saturated fatty acids, monounsaturated and polyunsaturated acids did not lead to TLR4 signal activation. Moreover, cell pretreatment in vitro for three hours with different polyunsaturated fatty acids, particularly the ω-3 fatty acid docosahexaeanoic acid (DHA: 22: 6 ω-3), or oleic acid (ω-9) significantly reduced the subsequent pro-inflammatory effect induced by lauric acid [103]. 

Saturated fatty acids represent an essential component of bacterial endotoxins. The lipid A portion of LPS has six saturated fatty acids coupled to this structure through ester or amide bonds. The carbon chain length of these fatty acids in lipid A varies from 12 to 16 carbons. Interestingly, the replacement of these saturated fatty acids by monounsaturated or polyunsaturated fatty acids stops the pro-inflammatory activity of the LPS [104]. 

Saturated fatty acids can also induce an inflammatory response through the activation of TLR2, which forms heterodimers in the plasma membrane, along with TLR1 or TLR6. Diacylated and triacylated lipoproteins, peptidoglycans, and lipoteichoic acid are among this receptor’s agonists [76,105,106]. Lee et al. [107] reported that lauric acid induced activation through NF-κB when TLR2 was cotransfected with TLR1 or TLR6; however, this did not occur when TLR1, 2, 3, 5, 6, or 9 were individually transfected. On the other hand, the omega-3 polyunsaturated fatty DHA suppresses activation through the NF-κB signaling pathway, whether this is induced by LPS or by lauric acid [108]. Furthermore, the inhibition of TLR2 expression enhances the sensitivity to insulin action in the skeletal muscle and in the white adipose tissue of mice that were fed on a high fat diet as well as inhibiting the expression of this receptor. This process results in the partial reversal of palmitic acid-induced insulin resistance [23,109].

Erridge and Samani [110] suggested that saturated fatty acids would not directly stimulate TRL2 and TLR4, but that this effect could result from the contamination of the bovine serum albumin that was used to solubilize the saturated fatty acids in the studies conducted in vitro. However, Huang et al. [76] demonstrated that saturated fatty acids activate the inflammatory response in vitro through TLR2 and TLR4. Lauric acid—which was not solubilized in bovine serum albumin—induced the activation of the NF-κB signaling pathway through TLR2—which was dimerized with TLR1 or TLR6—and TLR4. In addition, there are current propositions addressing TLR4 activation by saturated fatty acids that depend on fetuin A, which is produced in the liver and works through endogenous TLR4-binding [77]. 

Palmitate acid that is bound to TLR4 activates the kinase proteins JNK and IKK-β, and increases the expression and secretion of pro-inflammatory cytokines [86]. Palmitic acid also impairs insulin signaling pathways by inducing IRS-1 phosphorylation at serine residue position 307 [111]. This process reduces its interactions with the insulin receptor, and, consequently, diminishes the insulin-induced signal transduction. Moreover, saturated fatty acids induce insulin-action resistance due to the antagonistic action of the peroxisome proliferator-activated receptor-gamma coactivator (PGC)-1 alpha. Such a process induces the expression of mitochondrial genes that are involved with oxidative phosphorylation and with glucose capture, which is mediated by insulin [112,113]. 

### 4.2. Polyunsaturated Fatty Acids

Polyunsaturated fatty acids consist of two families (ω-3 and ω-6) that are characterized by the double bond locations defined by the first double bond in relation to the methyl terminal group in the fatty acid molecule. α-Linolenic and linoleic acids are examples of polyunsaturated fatty acids belonging to the ω-3 and ω-6 families, respectively. These two fatty acids are not synthesized in humans, and the lack of ω-3 and ω-6 intake causes signaling and symptom deficits, indicating that such nutrients are essential to humans; therefore, they must be consumed through the diet [24,25,114,115]. However, studies have shown that the ratio of ω-6 to ω-3 fatty acids in the diet has implications for health since increased ratios are associated with an increased risk of chronic disease incidence and progression [116,117]. 

α-Linolenic acid is the precursor of the ω-3 polyunsaturated fatty acids with a longer chain and a high degree of unsaturation, such as eicosapentaenoic acid (EPA: 20: 5 ω-3) and DHA, which are found in seafood, especially fatty fish, and in fish oil supplements. It is important to note that the α-linolenic concentration in the blood, cells, and tissues is significantly lower than that of the EPA and DHA. This suggests that the primary biological function of α-linolenic is as a substrate in EPA and DHA synthesis [118]. However, evidence shows that α-linolenic conversion into EPA and DHA in humans is relatively low: conversion into EPA is estimated to only be around 8–12%, and conversion into DHA is lower than 1% [119,120]. 

The beneficial effects resulting from an increased intake of ω-3 fatty acids were originally associated with the suppression of thrombosis. However, epidemiologic evidence suggests that the intake of ω-3 fatty acids reduces the morbidity and mortality rates due to cardiovascular diseases, as well as reducing systemic blood pressure, triacylglycerol concentrations, and the risk of endothelial dysfunction [27,121,122,123,124,125,126]. The capacity to lower triacylglycerol concentrations, which is related to diminished hepatic VLDL secretion, stands out among the aforementioned possible metabolic effects resulting from the intake of ω-3 fatty acids. This effect is partially dependent on mechanisms that are related to nuclear receptors, particularly the peroxisome proliferator activated receptor (PPAR)-α [127].

An increased intake of ω-3 fatty acids results in the corresponding accumulation of these fatty acids in cell membranes and circulating lipids. They replace ω-6 fatty acids (such as linoleic and arachidonic acids) in blood lipids and in cell membranes, and also modulate/activate different signaling pathways [128]. 

The ω-3 and ω-6 polyunsaturated fatty acids generate relevant modulations in the inflammatory response because they are precursors to different series of eicosanoids, which have different effects on the intensity of the inflammatory response. Accordingly, ω-6 arachidonic acid generates even-series eicosanoids, such as prostaglandin E_2_ and leukotriene B_4_. These eicosanoids induce pro-inflammatory effects, such as increased vascular permeability, vasodilation, fever, and chemotaxis. It is important to note that prostaglandin E_2_ also has anti-inflammatory effects, such as reduced IL-1 and TNF-α production. EPA is the precursor for odd-series eicosanoids, such as prostaglandin E_3_, thromboxane A_3_ and leukotriene B_5_, which induce lower-intensity inflammatory responses. Leukotriene B_5_, for example, is 10 to 100 times less potent as a chemotactic agent in neutrophils than leukotriene B_4_ [23,27,129]. EPA also competes with arachidonic acid for COX-2 and 5-LOX; therefore, EPA reduces the synthesis of even-series eicosanoids [130]. In addition, higher EPA and DHA concentrations in the plasma membrane favor the production of mediators, such as resolvins, maresins, and protectins, which are involved in the resolution of inflammation and healing [21,25,131]. 

The ingestion of alpha-linolenic acid can also modulate the inflammatory response in humans. For example, Caughey et al. [132] observed a significant reduction of TNF-α, IL-1β, TXB_2_, and PGE_2_ production by LPS-stimulated mononuclear cell cultures that were obtained from healthy subjects who consumed approximately 14 g/day alpha-linolenic acid for four weeks as compared to baseline and to a control group. The effect of α-linolenic acid may have been mediated through its conversion to EPA.

With regard to the molecular effects of EPA and DHA on inflammatory-response modulation, studies have shown that these fatty acids inhibit the expression of inflammatory genes, such as COX-2, iNOS, and IL-1 in macrophages [103,108]. In contrast to the stimulating effect of saturated fatty acids on TLR2 and TLR4 activation, EPA and DHA are capable of mitigating the activation of the NF-κB transcription factor pathway that is induced by various agonists [103,133,134]. Thus, DHA reduces NF-κB pathway activation and the expression of cytokines and COX-2 induced by TLR agonists, such as lipopeptides (TLR2) and LPS (TLR4) in macrophages [89]. In addition, there is reduced gene expression of COX-2 that is induced by LPS in monocytes from the peripheral blood of individuals who use fish oil supplements [103,108]. The synthesis of the cytokines IL-1, IL-2, and TNF-α was also mitigated after stimulation with LPS in vitro by mononuclear cells from the peripheral blood from individuals that were supplemented with 18 g of fish oil per day for six weeks [135]. 

In addition, EPA and DHA present another mechanism to modulate the inflammatory response by binding to G-protein coupled receptor 120 (GPR120), which is also known as free fatty acid receptor 4 (FFA4). GPR120 activation induced by EPA or DHA leads to β-arrestin 2 recruitment to the plasma membrane, where this protein binds to GPR120. Subsequently, the GPR120/β-arrestin 2 complex is internalized into the cytoplasmic compartment, where this complex binds to the TAK1-binding protein (TAB1). This process impairs the association between TAB1 and the kinase activated by the growth factor beta (TAK1), and, consequently, results in reduced TAK1 activation and in reduced activity of the IKK-β/NF-κB and JNK/AP-1 signaling pathways. Accordingly, the TAB1/TAK1 binding is a convergence point of stimuli that are induced by the TLR4 signaling pathway and of the TNF receptor (TNFR). The mitigation of TAK-1 activation by DHA leads to the reduced expression of genes with pro-inflammatory actions, such as TNF-α and IL-6 [136,137]. 

Other mechanisms that are related to the EPA and DHA effects concern their capacities to bind to peroxisome proliferator activated receptors (PPARs), including the isoforms PPAR-alpha, PPAR-gamma, and PPAR-beta/delta. PPARs are a group of nuclear receptors that are coded for by different genes. PPAR isoforms form heterodimers with the retinoid X receptor (RXR) and bind to peroxisome proliferator response elements (PPRE) in the region that is responsible for promoting the target genes that are involved in lipid metabolism and in the inflammatory response; subsequently, they modulate the expression of these genes [138]. PPAR-alpha and PPAR-gamma activations reduce the expression of genes that code for proteins presenting pro-inflammatory actions through inhibition of NF-κB activation. It is worth emphasizing that EPA and DHA directly interact with PPARs, and, therefore, modulate the expression of genes that are involved in lipid metabolism and the inflammatory response [139]. Furthermore, the anti-inflammatory effects of EPA and DHA on this signaling pathway can occur due to diminished nicotinamide adenine dinucleotide phosphate (NADPH) oxidase activity, which leads to lower TLR4 recruitment for lipid rafts and TLR4 dimerization [102]. Moreover, the lower NADPH oxidase activity also decreases the production of reactive oxygen species, which, in turn, are necessary to activate the TLR4 signaling pathway. Another possible mechanism of action of the ω-3 fatty acids concerns the capacity of incorporating DHA into the plasma membrane, which can lead to reduced TLR4 translocation for lipid rafts formation. This decreases TLR4 pathway activation, and, consequently, decreases NF-κB activation [102,140,141]. 

**Figure 3:**
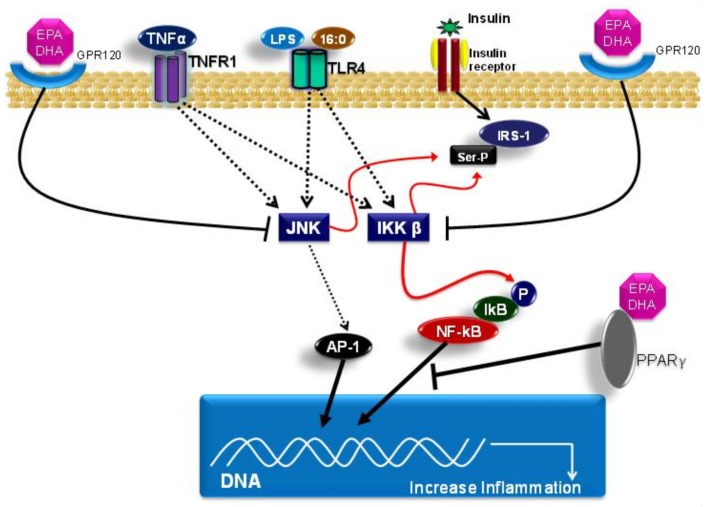
Molecular mechanism of the effects of saturated (16:0) and omega-3 polyunsaturated fatty acids (EPA, DHA) on the TLR4 and NFkB pathways. The arrows → indicate activation and the arrows Ⱶ indicate inhibition. Abbreviations: TNFα, Tumor necrosis factor*;* TNFR1, Tumor necrosis factor receptor 1; LPS, Lipopolysaccharides; 16:0, palmitic acid; TLR4, Toll-like receptor 4; GPR120, G-protein coupled receptor 120; EPA, eicosapentaenoic acid; DHA, Docosahexaenoic acid; IRS-1, Insulin receptor substrate 1; Ser-P, phosphorylated *serine* residues; PPARγ, Peroxisome proliferator-activated receptor gamma; JNK, c-Jun N-terminal kinases; IKK β, inhibitor of nuclear factor kappa-B kinase subunit beta; IkB, NFKB Inhibitor; P, phosphate; AP-1, Activator protein 1.

Figure 3 shows the main molecular mechanisms related to the effects of saturated and omega-3 fatty acids on the TLR4 pathway. 

## 5. Conclusions

The inflammatory process that occurs in obese people differs from the classical inflammatory response in certain respects. This inflammatory process manifests itself systemically and is characterized by a chronic low-intensity reaction. In this context, the TLR4 signaling pathway has been recognized as one of the main triggers in increasing the obesity-induced inflammatory response. This pathway responds to the increased exposure to saturated fatty acids and to LPS. Both of these are relevant in the context of obesity, with saturated fatty acids arising from within the adipose tissue triglyceride stores and the LPS arising from increased intestinal permeability perhaps due to an altered gut microbiota. Adipose tissue driven inflammation increases insulin resistance, both locally and systemically, so contributing to the co-morbidities of obesity, like DM2. Studies indicate that omega-3 fatty acids, namely EPA and DHA, have an anti-inflammatory effect, which involves attenuating the activation of the TLR4 signaling pathway. This has relevant implications for reducing meta-inflammation, and, consequently, resistance to insulin action and the risk of DM2 and cardiovascular disease in obese individuals. The omega-3 fatty acids can oppose the action of both classic TLR agonists (e.g., LPS) and saturated fatty acids in this regard.
